# The unmet need for pertussis prevention in patients with chronic obstructive pulmonary disease in the Italian context

**DOI:** 10.1080/21645515.2019.1652517

**Published:** 2019-09-06

**Authors:** Francesco Blasi, Paolo Bonanni, Fulvio Braido, Giovanni Gabutti, Federico Marchetti, Stefano Centanni

**Affiliations:** a Department of Pathophysiology and Transplantation, University of Milan, Milan, Italy; b Department of Internal Medicine, Respiratory Unit and Adult Cystic Fibrosis Center, Fondazione IRCCS Ca’ Granda Ospedale Maggiore Policlinico, Milan, Italy; c Department of Health Sciences, University of Florence, Florence, Italy; d Department of Internal Medicine and Medical Specialties, IRCCS Ospedale Policlinico San Martino, Genoa, Italy; e Department of Internal Medicine and Medical Specialties, University of Genoa, Genoa, Italy; f Department of Medical Sciences, University of Ferrara, Ferrara, Italy; g Vaccines Medical Department, GSK, Verona, Italy; h Respiratory Unit, Department of Health of Sciences, ASST Santi Paolo e Carlo, University of Milan, Milan, Italy

**Keywords:** COPD, pertussis, whooping cough, Italy, vaccination, Tdap vaccine

## Abstract

Despite high rates of vaccination, pertussis resurgence has been reported worldwide in recent years, including in Italy, especially in older adults.

Chronic obstructive pulmonary disease (COPD) is a respiratory disease associated with progressive inflammation of the respiratory tract. Regional population studies have shown the prevalence of COPD in Italy to be approximately 15% with an age-dependent increase in proportion of COPD cases.

Emerging data shows that individuals with COPD are at high risk of contracting pertussis. Furthermore, those who develop pertussis could experience exacerbation of their pre-existent COPD and further susceptibility to other infections.

Immunization programs in Italy currently recommend a decennial reduced-antigen-content diphtheria-tetanus-acellular pertussis booster vaccine dose for adults. Active measures to encourage booster vaccination, especially for high-risk adults such as those with COPD, could positively impact pertussis morbidity and the associated healthcare burden.

## Introduction and aim

Pertussis is a highly contagious respiratory disease caused by the bacterium *Bordetella pertussis* (*B. pertussis*) and transmitted directly from the infected to the susceptible subject.^1–3^ Following attachment to the cilia of the respiratory epithelial cells, the bacterium produces toxins that have systemic effects on immune system and locally paralyze the cilia and cause inflammation of the respiratory tract, which subsequently interferes with the clearing of pulmonary secretions.^4^ Until recently, *B. pertussis* was considered to be a non-invasive pathogen; however, recent studies have shown that the bacteria can enter certain cells of respiratory origin such as alveolar macrophages^5^ and epithelial cells.^6^

Pertussis in children is characterized by an initial catarrhal phase, presenting symptoms such as mild fever, runny nose and cough which appear 7–10 days after infection. In typical cases, the disease gradually progresses to the paroxysmal phase where pertussis patients exhibit the distinct “whooping cough”. The subsequent convalescent phase of the disease may last for several months.^2,3^ In infants and children, pertussis can result in significant pathological complications, which can be classified into three categories: pulmonary, neurologic (acute pertussis encephalopathy), and nutritional.^7^ Moreover, in severe cases, extreme lymphocytosis could evoke intractable pulmonary hypertension, respiratory failure and ultimately, death.^5^

In contrast, in adults, pertussis presents in an atypical manner without the classical symptoms, rendering pertussis diagnosis challenging, and is usually manifested as a protracted cough and lesser severity in those who have been previously vaccinated during childhood.^8^ Among adolescent and adult patients, pertussis complications such as sinusitis, otitis media, urinary incontinence, pneumonia, weight loss, rib fracture, and fainting have been described.^9^

**Figure 1.**
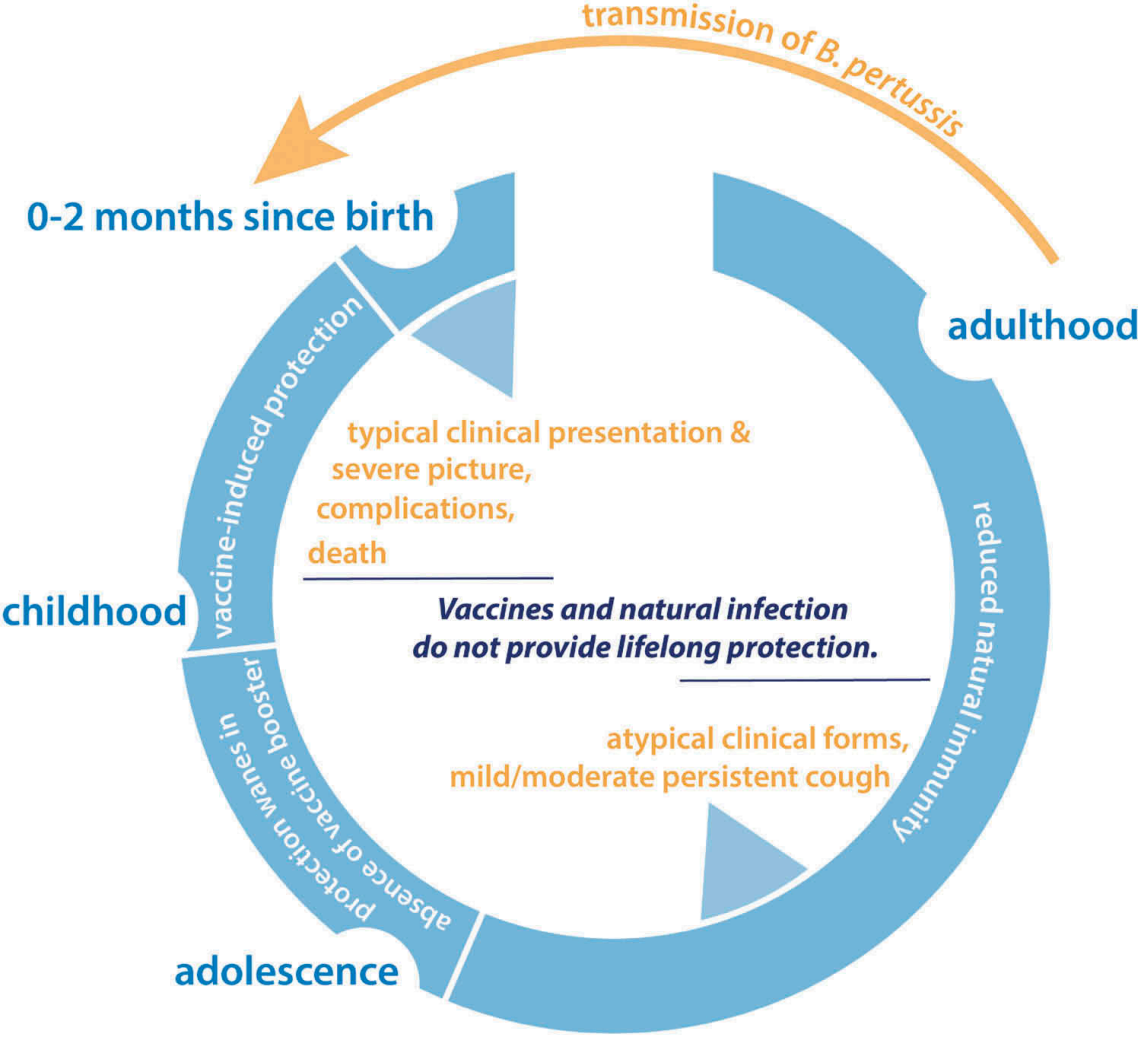
The pertussis cycle. *B. pertussis, Bordetella pertussis* The reduction of natural immunity in adulthood not only renders people susceptible to pertussis infections but also makes them reservoirs of *Bordetella pertussis* which can transmit the infection to the unvaccinated population.^12^

Pertussis was widespread in the pre-vaccination era, mainly affecting children between the age of 1–9 years.^10^ However, the introduction of whole-cell pertussis (wP) vaccines in the late 1940s resulted in a substantial decline in the incidence of pertussis and its associated mortality.^3^ In the 1990s, the wP vaccines were replaced by the more defined monocomponent or multicomponent acellular pertussis (aP) vaccines, with an intention to improve safety and reduce the number of side effects.^3^ Neither the natural infection by *B. pertussis* nor vaccines against pertussis, wP or aP, provide lifelong immunity against pertussis and healthcare authorities have noticed an increase in the number of pertussis cases and epidemic cycles worldwide in recent years.^1^ This strongly indicates that despite a high level of vaccine coverage, the global threat posed by *B. pertussis* infections is not adequately controlled yet. The increasing incidence of pertussis among older children, adolescents and adults, especially those who are >65 years of age or with comorbidities and pathological conditions such as chronic obstructive pulmonary disease (COPD) is of particular concern. These infected populations serve as a source of transmission to young infants who are incompletely immunized or not immunized at all and are more prone to pertussis-related complications and even death.^11,12^ (Figure 1).

In order to counter this threat, certain Western countries have implemented 10-year booster vaccine programs against pertussis^13^ and health organizations such as the Center for Disease Control and Prevention (CDC), Public Health England, and the Global Pertussis Initiative (GPI) recommend immunization with the reduced-antigen-content diphtheria-tetanus-acellular pertussis (Tdap) vaccine for pregnant women as well as others who could be in contact with newborn children.^14–16^

When taking into consideration the risk of pertussis infections in adults, especially those aged 65 years and above, particular attention should be reserved for those with chronic respiratory diseases. COPD is a chronic pathology of the respiratory system, depicted by persistent and partially reversible airflow obstruction as a result of pulmonary remodeling of the bronchioles (small airway disease), the bronchi (chronic bronchitis), and the lung parenchyma (pulmonary emphysema).^17,18^ The disease presents a substantial burden on quality-of-life (QoL) and healthcare resources and is projected to be the third leading cause of death worldwide by 2030.^17^ The prevalence of COPD in Europe has been reported to be approximately 12% in 2010^19^ with a matching prevalence in Italy.^20,21^ Results from an international study published in 2003 reported COPD to be prevalent in 15% in Italian adults aged 45–97 years.^20^ More recently, a multicentre study conducted across Italy showed an age-dependent prevalence of COPD, ranging from 3.3% in adults aged 20–44 years to 13.3% in those aged 65–84 years.^21^

COPD is often a result of long-term external damages to the respiratory system, either through involuntary exposure to environmental pollutants or voluntary lifestyle practices such as smoking. The sustained inflammation of airways leads to the destruction or loss of lung tissue (emphysema) and scarring (remodeling and fibrosis).^22^ The inflammatory response in COPD is modulated by the immune system (migration of T and B cells, neutrophils, and macrophages to the lung) and pro-inflammatory cytokines (tumor necrosis factor-alpha, interferon-gamma, interleukin [IL]-1, IL-6, IL-8, IL-17, IL-18, and IL-32).^23^ In addition to a weakened immune system, as a result of receiving immunosuppressive therapies (corticosteroids) in the long term, COPD patients also have structural modifications of the respiratory tract with inflammation and hyper-production of mucus, defects in the load of the ciliated epithelium, and a deficiency in innate and acquired immunity.^24^

This review aims to inform healthcare professionals, patients and the general population on the possibility of COPD being a risk factor for pertussis and on the value of the Tdap booster vaccination in adults and older adults with COPD. To prepare this manuscript, a non-systematic, narrative literature review was carried out. References were mainly retrieved from PubMed and Embase up to February 2019 by combining the following keywords: pertussis, whooping cough, COPD, adults, vaccination, Tdap vaccines, Italy, diphtheria, tetanus. Other technical or scientific documents were retrieved from Google searches.

## Potential relationship between COPD and host immunity

Besides its impact on QoL, productivity, and healthcare costs, COPD is associated with various comorbidities – lung cancer, cardiovascular disease, hypertension, and diabetes.^25^ However, the most significant issue in patients with COPD is their susceptibility to bacterial and viral infections.^24^ Under normal circumstances, the epithelial lining of the respiratory tract is an efficient barrier against infectious agents despite persistent exposure. However, various molecular and cellular processes of COPD lead to pathologic changes, especially in the mucociliary function, which results in the disruption of this innate defense mechanism.^24^

One of the main causative factors of COPD is long-term cigaret smoking.^26^ The effect of smoking on the elevated susceptibility of smokers to respiratory tract infections of bacterial and/or viral etiology is well documented. Various reports since the population-based Tecumseh study published in 1975^27^ have demonstrated a higher risk of developing acute respiratory infections in patients with COPD who were also smokers;^28^ cigaret smoking has also been implicated in pathogen-induced exacerbation of COPD.^29^ Cigaret smoke has been postulated to exert its immunosuppressive effects via altered mucociliary function and gap junctions of epithelial cells; impaired functionality of alveolar macrophages, dendritic cells, and natural killer cells; neutrophil-mediated epithelial cell damage; and lowered humoral and cell-mediated immunity.^30^ In addition, it is also proposed that cigaret smoke increases pathogenic virulence and antibiotic resistance.^30^ Hence, smoking constitutes a significant risk factor for infections in COPD patients.^31^

Episodes of exacerbations in COPD lay a huge burden, not only on morbidity and mortality of the disease, but also on healthcare resources, and are mainly a result of microbial infections.^24,31^ Moreover, microbial colonization of the pulmonary tissue further increases the inflammatory burden and accelerates disease progression in COPD.^24^

## Clinical evidence on the association of COPD and pertussis

While the association between COPD and pertussis in clinical settings has not been investigated extensively, some preliminary studies have shown, albeit in a non-definitive manner, an increase in the risk of pertussis in patients with COPD as compared to non-COPD patients.^32,33^

In their pilot study, Bonhoeffer et al.^32^ assessed patients with acute exacerbations of chronic bronchitis (AECB) (N = 26) for a concomitant presence of *Bordetella* infection using nasopharyngeal swabs for culture, polymerase chain reaction, and blood samples for serology. The investigators discovered that eight of the patients (31%) were seropositive for *Bordetella* infections, five of them (19%) being confirmed by ELISA technique to be due to *B. pertussis*.^32^

Following a case–control study comparing the seroprevalence of *B. pertussis* in 90 patients with COPD with an equal number of control patients without COPD, Hashemi et al.^33^ reported no significant association between COPD and anti-pertussis toxin (PT) IgA seropositivity (*p* = 0.448), indicative of recent infection. In contrast, they observed a statistically significant association between COPD and anti-PT IgG seropositivity (*p* < 0.001), implying a history of infection and which could be indicative of persistent subclinical infections. While the investigators could not discern any association between *B. pertussis* seroprevalence and severity of COPD, the colonization by *B. pertussis* could serve as a potential risk factor for the development of COPD.^33^

These preliminary findings which hint at an association between *B. pertussis* and COPD were also confirmed in a retrospective cohort study conducted in the United States (US) which aimed at ascertaining the incidence and economic burden of diagnosed pertussis in individuals ≥11 years with COPD or asthma. The study analyzed records from a large group of eligible patients who had been diagnosed with pertussis and had pre-existing asthma or COPD and compared these to records from individuals diagnosed with pertussis but without asthma or COPD.^34^

The salient findings of this study^34^ on the number of diagnosed pertussis among adolescents and adults with pre-existing COPD or asthma are summarized in Table 1.

**Table 1.** Summary of the findings from a United States retrospective cohort study evaluating the incidence and economic burden of diagnosed pertussis in individuals with COPD or asthma.^34^

a) Incidence of diagnosed pertussis among adolescents and adults with pre-existing COPD or asthma				
Cohort	N	Sum of follow-up time (years)	Incidence per 1,000 person-years (95% CI)	Relative risk vs. patients with no pre-existing COPD (95% CI)
Patients with pre-existing COPD and pertussis	1,313	1,940	**0.176** (0.166–0.185)	**2.533** (2.396-2.678)
Patients with pre-existing COPD and no pertussis	2,681,930	7,476,119		
Patients with no pre-existing COPD or asthma and pertussis	20,672	48,521	**0.069** (0.068–0.070)	−
Patients with no pre-existing COPD or asthma and no pertussis	124,228,622	298,178,050		
b) Difference in likelihood of hospitalizations after pertussis diagnosis				
		COPD + pertussis cohort (N = 343)	Matched cohort (N = 343)	Difference
All cause ≥1 hospital admission (%)^a^		12.24	6.41	5.83
Pertussis-related ≥1 hospital admission (%)^b^		4.08	2.33	1.75

*CI*, *confidence interval;* *COPD*, *chronic obstructive pulmonary disease;* *N*, number.

*COPD*, chronic obstructive pulmonary disease; *N*, number.

^a^All-cause hospitalizations reflect the difference in the percentage of patients with hospitalization during the 45-day period post-pertussis index date (date of the first observed pertussis diagnosis minus 15 days) relative to the 45-day period pre-pertussis index date.

^b^Pertussis-related hospitalizations reflect the percentage of patients with hospitalization related to pertussis in the 45 days post-pertussis index date (date of the first observed pertussis diagnosis minus 15 days)

The difference in the likelihood of hospitalization after pertussis diagnosis is depicted here as follows:

- The likelihood of being hospitalized, irrespective of the duration of the observation period, was higher in patients with COPD than in controls.
- Patients with diagnosed pertussis and pre-existing COPD accrued significantly (*p* < 0.0001) more healthcare costs than controls (patient without a diagnosis of COPD).

In a study conducted among 464 hospitalized pertussis case-patients in seven US states in 2010–2014, 165 reported at least one underlying condition on past medical history, 89.2% of them being ≥21 years old and 31.4% with a history of asthma and/or COPD.^35^

Lastly, the findings from a recent study led by authors from CDC, which evaluated severe pertussis infections across various states in the US during the period 2011–2015, showed that 14.5% of patients aged 21–64 years and 26.8% of those ≥65 years who were hospitalized for pertussis also presented with COPD indicating that underlying COPD may contribute to the clinical severity of pertussis infections.^36^

Age-related changes in adaptive and humoral immunity in adults result in immunosenescence, i.e., a diminished immune response against certain diseases.^37^ These changes can not only result in infections in such susceptible adults but also result in them becoming reservoirs of the causative agents, infecting those who cannot be vaccinated. Pertussis is still endemic and outbreaks have been reported.^38^ Morbidities such as COPD are known to result in susceptibility to infectious agents which in turn can lead to episodes of exacerbations of COPD in these patients. Moreover, since it has been reported that pertussis protection is not lifelong unless a vaccine booster is given,^39^ it could be postulated that specific disease-susceptible populations such as those presenting with COPD, could be especially prone to pertussis infections.

**Figure 2.**
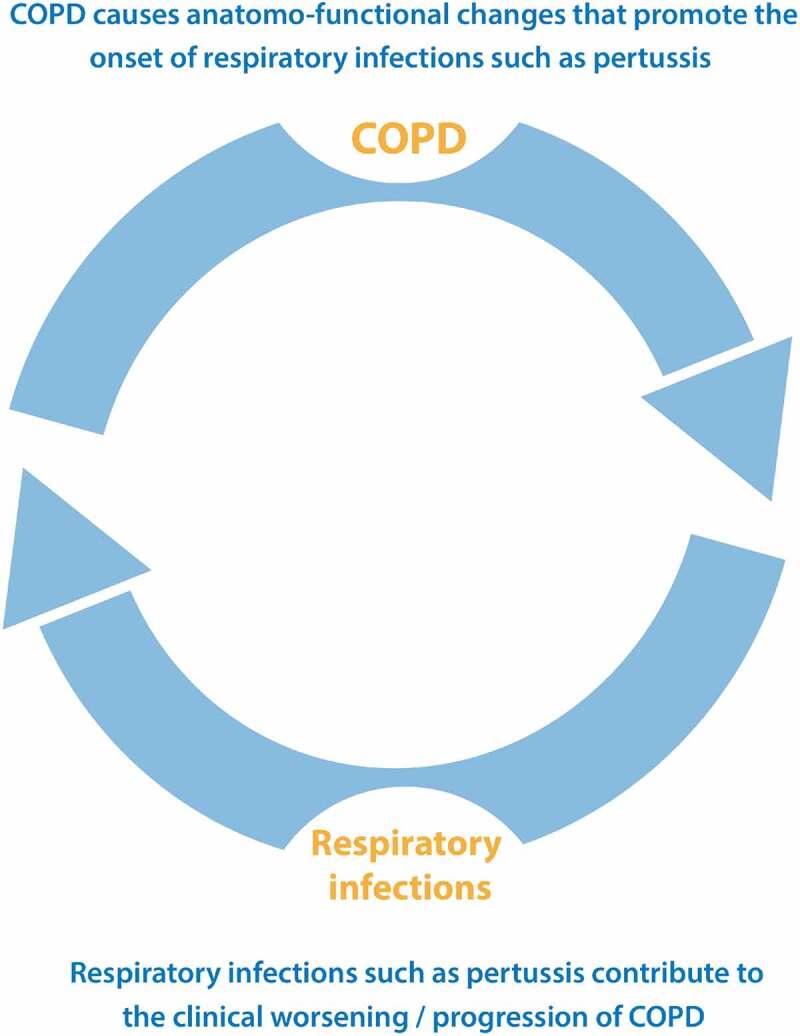
Correlation between respiratory infectious episodes and COPD. *COPD*, chronic obstructive pulmonary disease. Depicted is a cyclic relationship between COPD and respiratory infections such as pertussis. The presence of COPD renders the patient susceptible to infection by *Bordetella pertussis* which in turn can contribute to the exacerbation of COPD.

In other words, one can hypothesize a type of vicious cycle, where the chronic disease promotes the onset of the infection, in this case by *B. pertussis*, and where the infection itself promotes the progression of the chronic disease^40^ (Figure 2).

## Existing international recommendation on Tdap booster in COPD patients

On the basis of the COPD patient’s susceptibility to infections and the continually evolving clinical evidence, the Global Initiative for Chronic Obstructive Lung Disease (GOLD)^41^ and the CDC have recommended vaccination schedules in patients with chronic respiratory disease.^40^ Thereby, seasonal influenza and pneumococcal vaccination are already recommended for COPD patients.^40,41^ Findings from population-based studies suggested that COPD patients, particularly the older adults, had decreased risk of cardiac comorbidities when they were vaccinated by these vaccines.^42,43^ Moreover, an analysis of the effectiveness of influenza and pneumococcal vaccination in COPD patients in reducing the incidence of influenza-related illness and pneumococcal pneumonia showed that the incidence of adverse events was comparable between the vaccinated and control groups, thereby establishing an acceptable safety profile for both vaccines.^44^

For pertussis prevention, the CDC recommends a single dose of Tdap for adults aged 19 years or older who have not received a previous Tdap vaccine, and this also applies to patients with chronic respiratory disease.^40,45^ In Europe, the European Center for Disease Prevention and Control (ECDC) has made adult vaccination recommendations.^46^ GOLD guidelines have not yet incorporated a specific recommendation of Tdap booster in COPD patients.^41^

## Pertussis, diphtheria, and tetanus in italy

As the decennial Tdap dose allows for simultaneous boostering of the immunity against pertussis, diphtheria, and tetanus, the Italian epidemiology of the three diseases is briefly reviewed in this section.

Recent data published by the Istituto Superiore di Sanità (National Institute of Health), technical body of the Italian Ministry of Health, shows a resurgence of *B. pertussis* with an increased incidence not only in infants and children^47^ but also in adults and older adults in Italy.^48^ Comparison of seroprevalence data from two collection periods – 1996–1997 and 2012–2013 – shows a substantial increase in the proportion of seropositive people, especially in those >60 years of age (38.8% vs. 18.6%; *p* < 0.0001).^48^

Disease under-recognition and under-notification is a major hindrance to the awareness of the real burden of pertussis.^47^ Pertussis has been identified by the Istituto Superiore di Sanità (National Institute of Health) as an emerging problem in Italy. Hence, the 2017–2019 Italian National Immunization Prevention Plan (Piano Nazionale di Prevenzione Vaccinale, PNPV) has made provision for pertussis boosters for adolescents as well as for adults and older adults every 10 years.^49^ This measure aims to achieve individual protection as well as to reduce *B. pertussis* circulation and transmission to unvaccinated population such as infants.^48^

The causative agents behind diphtheria and tetanus are the bacteria *Corynebacterium diphtheriae* and *Clostridium tetani*, respectively.^50,51^ Whereas *Corynebacterium diphtheriae* is transmitted from person to person or via cutaneous diphtheria lesions,^50^ *Clostridium tetani* enters the human body through open wounds or tissue injuries that come in contact with materials infected with *Clostridium* spores.^51^

Diphtheria and tetanus pathologies are mediated by their respective bacterial toxins. Since tetanus is directly acquired from the environment, herd immunity is not possible although in some countries, maternal immunization protecting infants during the neonatal period is recommended.^51,52^

Serologic protection decreases with increasing age. In a seroprevalence study describing tetanus epidemiology in Italy from 2001 to 2010, it has been reported that less than 30% of subjects aged ≥65 years had protective tetanus antibody levels.^53^ Approximately 50 cases of tetanus are notified in Italy every year, of which approximately 80% are in the subjects aged ≥64 years, mostly women, and with an average number of 21 deaths per year.^53^

For diphtheria, immunity depends on presence of antibodies to the diphtheria toxin at the time of infection and cell-mediated immunity may also contribute. For neonates, maternal antibodies delivered through the placenta could provide passive immunity for the first few months.^50^ Diphtheria seroprevalence data collected from eight Italian cities in the mid-90s has revealed a progressive reduction in the proportion of subjects with protective anti-diphtheria antibody titers with increasing age and approximately one-third (33.4%) of subjects >60 years of age had antitoxin levels below protective concentrations.^54^ A more recent study conducted in Austria discovered that 65% of subjects >60 years of age did not have protective diphtheria antibody titers at the time of receiving a booster vaccine. Five years after booster vaccination, this percentage further declined to 45%.^55^

## Tdap booster recommendations in italy

PNPV 2017–2019 is a guidance document which aims at harmonizing immunization policies across Italy. Italian health authorities have addressed the call for action by the WHO, the Council of European Union (EU), and ECDC to promote and support vaccination policies to overcome the growing “vaccine hesitancy”.^56^ As one of the strategies to achieve this, PNPV allows for vaccines to be actively offered free-of-charge to target populations.^57^

One of the novel policies adopted by PNPV is the recommendation of the Tdap vaccine for adults and older adults, every 10 years.^49^ More precisely, the novelty lies in the addition of the pertussis component to the diphtheria-tetanus vaccine (Td) previously recommended.

The Italian Ministry of Health has recently reiterated that all the vaccinations included in the national schedule were to be included in the new Essential Levels of Assistance (LEA in Italian), i.e., healthcare assistance which is guaranteed to every Italian citizen. This mandate further states the obligation of the Regions to offer for free vaccinations, which, although not mandatory, are provided for in the PNPV.^58^

Tdap vaccines are available, recommended, help to protect against three diseases simultaneously, and are generally well tolerated.^59^ As a support for healthcare providers, the Guide of Contraindications to Vaccination,^60^ produced by eminent experts in vaccinology, in collaboration with national institutions in Italy (Ministry of Health, Italian Institute of Health, National Drug Agency, etc.), highlights that the contraindications to Tdap vaccination in adult and older adults are only confined to a few conditions (Table 2),^60^ thereby making a strong case for vaccinating COPD patients against pertussis.

**Table 2.** General or transient contraindications to Tdap booster.^60^

Diphtheria (Pediatric and Adult)		Tetanus	Pertussis (Adult)	
Contraindications	Temporary contraindications	Contraindications	Contraindications	Temporary contraindications
Severe allergic reaction (anaphylaxis) after administration of a prescribed dose.	Encephalopathy not attributable to another cause within seven days of receipt of a previous dose	Severe allergic reaction (anaphylaxis) after administration of a prescribed dose.	Severe allergic reaction (anaphylaxis) after administration of a prescribed dose.	Encephalopathy within seven days of administration of previous doses of aP (pediatric dosage) vaccine or ap (adult dosage) vaccine not attributable to other causes.
Severe allergic reaction (anaphylaxis) to a component of the vaccine.		Severe allergic reaction (anaphylaxis) to a component of the vaccine.	Severe allergic reaction (anaphylaxis) to a component of the vaccine.	

*Tdap*, reduced-antigen-content diphtheria-tetanus-acellular pertussis vaccine; *aP*, acellular pertussis (pediatric dosage); *ap*, acellular pertussis (adult dosage)

## The unmet need for Tdap booster vaccination in COPD patients in the italian context

In Italy, vaccination coverage figures are collected by the Ministry of Health for individuals aged ≤18 years.^61^ For those aged >18 years, the vaccination coverage is monitored for influenza only. In 2018, this coverage was of 52.7% for Italians ≥65 years old.^62^ In an Italian Respiratory Society web-based survey conducted among Italian pneumologists, 81% of the participants declared that they recommend influenza vaccination to their patients.^63^ However, a mean annual influenza vaccine coverage of only 20.2% (95% CI 18.4–22.1%) has been reported among Italian patients aged 18–64 years with chronic respiratory diseases in 2015–2018.^64^

Vaccination coverage for the other recommended pneumococcal and zoster vaccines among Italian ≥65 years of age were estimated to be around 30% and 14%, respectively.^65^

These low-reported coverages of recommended vaccines among older Italian adults, especially those with chronic respiratory diseases, and the lack of national pertussis vaccination coverage monitoring programs among this population suggest an unmet need of pertussis prevention among COPD patients in the Italian context.

The primary goal of promoting the decennial Tdap booster vaccination in adults and older adult patients with COPD is to protect this susceptible group from developing pertussis and thus to reduce disease transmission (Table 3).

**Table 3.** Objectives of decennial Tdap booster in patients with COPD.

Vaccinate the patient with COPD every ten years with Tdap to:
Help protect the patient from acute infectious episodes of pertussis
Help reduce the risk of hospitalization
Help reduce respiratory symptoms due to pertussis infection
Boosting immune response against tetanus and diphtheria
Potentially counter the spread of pertussis in the community (reducing the risk of pertussis in infants and family members)

*COPD*, chronic obstructive pulmonary disease; *Tdap*, reduced-antigen-content diphtheria-tetanus-acellular pertussis vaccine

This is particularly worth in countries like Italy where a resurgence of the circulation of *B. pertussis* among adults has been reported and where Tdap decennial vaccination promotion and coverage appears to be suboptimal.

**Figure 3.**
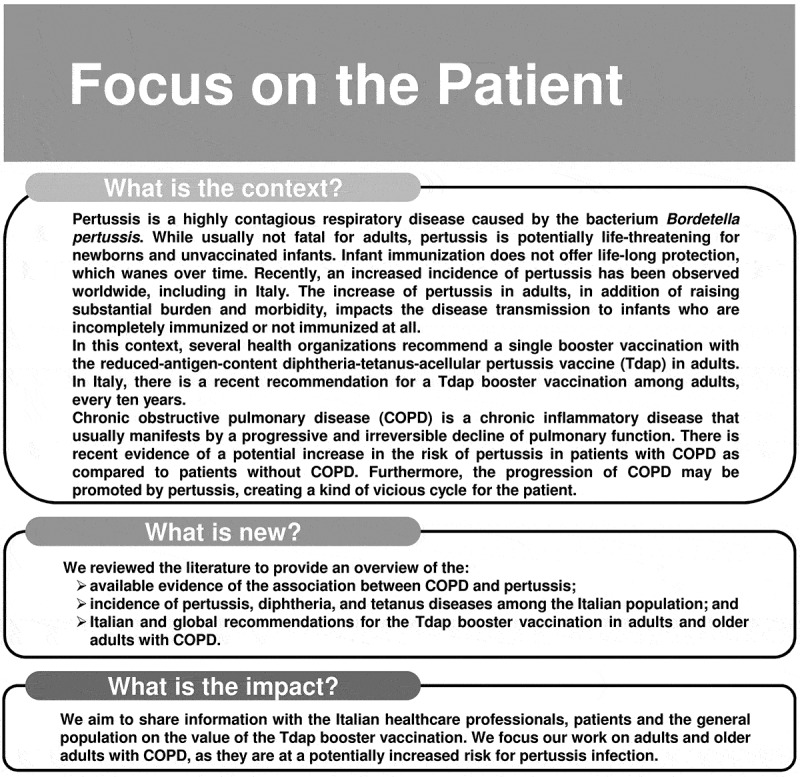
Focus on the patient.

On this basis, as summarized in Figure 3, the healthcare providers engaged in vaccination programs or taking care of COPD patients are encouraged to promote the value of Tdap decennial booster to achieve public health objectives stated by the PNPV and to protect COPD patients from pertussis and potential subsequent negative clinical worsening of their chronic disease.

### Contributorship

All authors participated in the literature review design or implementation or analysis, and interpretation of the literature review; and the development of this manuscript. All authors had full access to the data and approved the final manuscript. All authors agreed to be accountable for all aspects of the work in ensuring that questions related to the accuracy or integrity of any part of the work are appropriately investigated and resolved. The work described was carried out in accordance with ICMJE recommendations for conduct, reporting, editing and publications of scholarly work in medical journals. The corresponding author had the responsibility to submit the final manuscript for publication.
